# Adhesive Interfaces toward a Zero-Waste Industry

**DOI:** 10.1021/acs.langmuir.2c02436

**Published:** 2022-12-07

**Authors:** Adriana Sierra-Romero, Katarina Novakovic, Mark Geoghegan

**Affiliations:** School of Engineering, Newcastle University, NE1 7RU Newcastle upon Tyne, United Kingdom

## Abstract

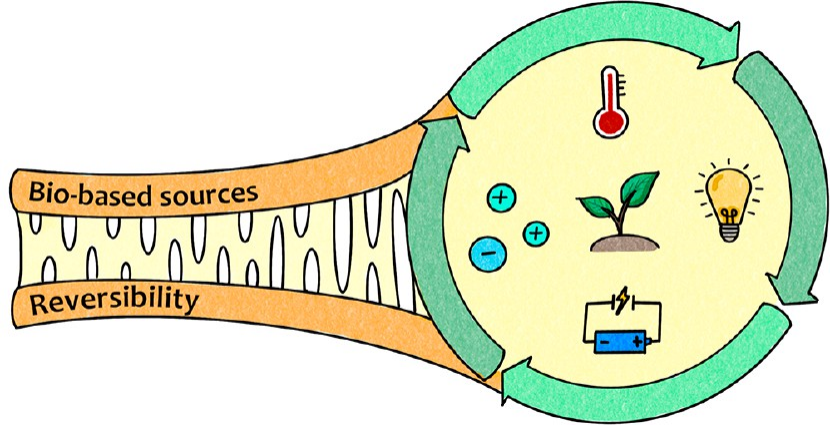

This Feature Article evaluates ongoing efforts to adapt adhesives toward the goal of zero-waste living and suggests the most promising future directions. Adhesives are not always considered in zero-waste manufacturing because they represent only a small fraction of a product and offer no additional functionality. However, their presence restricts the reintegration of constituent parts into a circular economy, so a new generation of adhesives is required. Furthermore, their production often leads to harmful pollutants. Here, two main approaches toward addressing these problems are considered: first, the use of natural materials that replace petroleum-based polymers from which conventional adhesives are made and second, the production of dismantlable adhesives capable of debonding on demand with the application of an external stimulus. These approaches, either individually or combined, offer a new paradigm in zero-waste industrial production and consumer applications.

## Introduction

According to the Global Waste Management Outlook from the United Nations Environment Programme and the International Waste Management Association, an estimate of 7 to 10 billion tons of waste is produced yearly worldwide with continued growth foreseen.^1^ The production of an increasing amount of solid waste has been a consequence of several factors including the manufacturing processes, the prevalence of single-use products (e.g., plastics), the population increase, and deficient waste management policies.^2^ The accumulation of these urban, commercial, industrial, and construction residues has created environmental and health hazards not only locally but also on a global scale.

To address the impact that waste has, the concept of a circular economy has become increasingly relevant. This implies that waste materials should return to the production chain through reusing or recycling, or if these are not possible, they should be used as a source of energy.^3^ Within the context of a circular economy, the term “zero waste” appears as the goal in which materials flow in a continuous circular system of production, use, and recovery, hence replacing the need for virgin materials and driving waste production to zero.^4^ The transition from a traditional industrial model to a zero-waste system implies the development not only of manufacturing technologies and policies but also of materials that can be readily reintegrated into the resource flows.

Adhesives are materials used in all types of manufactured products, and though their use is widespread, their presence is often underrated because they form a minimal weight percent of the product. Generally, adhesives are made of polymers as they possess various molecular structures that allow tailoring of their mechanical properties.^5^ However, they are mainly obtained from petroleum resources.^6^ In addition to polymers, adhesives contain chemicals that may be harmful or hazardous to workers, users, and the environment.

From a sustainability perspective, there is a need to integrate adhesives into a zero-waste system to reduce the environmental impact. From a manufacturing standpoint, this has to be accomplished by minimizing the effect on the adhesive properties. To achieve both requirements, a new generation of adhesives needs to be designed and produced.

Several guidelines have been defined on how to reduce the environmental impact of adhesives: the replacement of organic solvents, the replacement of solvent-based polymerization, surface treatment, solvent-free formulas, and biobased alternatives.^7^ However, achieving the formulation of greener adhesives is just a partial way to address the environmental issues as recent policies focus not only on environmental emissions but also on waste disposal.

The main challenge that adhesives represent within a circular economy and zero-waste industry is related to the source from which raw materials are obtained, recovery after use if the components are meant to resist separation, and how to reintroduce components into the production flow. This Feature Article provides an overview of the strategies that have been developed to meet the new environmental demands of adhesive interfaces and the research with the potential of leading the trends in this newly explored area.

## Brief Recap of Conventional Adhesives

The modern age of synthetic adhesives began during the second decade of the 20th century with the development of phenol formaldehyde adhesives^8^ and was consolidated during World War II. Though the first commercial adhesives were based on vegetable, animal, or mineral sources, as the chemical industry advanced and the product requirements became more specialized, synthetic adhesives appeared.^9^ Synthetic adhesives offer greater uniformity and control, making them more reliable than their natural counterparts.^10^ These are used widely, including, for example, across the packaging,^11^ textile,^12^ construction,^13^ and automotive^14^ industries.

Structural adhesives can be classified according to different categories.^15^ However, modern adhesives find their most useful categorization based on their hardening mechanism. Adhesives base their action on the premise that, once they have been applied, they must harden and strengthen through either cooling (e.g., molten thermoplastic), evaporating a solvent (e.g., water or organic solvent), or a chemical reaction (e.g., cross-linking). Pressure-sensitive adhesives appear as an independent adhesive classification as they remain permanently sticky.^16^

**Table 1:** Common Types of Adhesives (Adapted from Comyn^16^)

adhesive behavior	examples
cooling hardening	thermoplastic polymers such as poly(vinyl acetate), polyesters, cellulose derivatives, polyamide, phenoxy, and acrylics
solvent loss hardening	acrylics, rubbers (natural and synthetic), phenolics, polyurethanes, and vinyl resins
water loss hardening	natural adhesives (starch, casein, and cellulose derivatives), rubbers (natural and synthetic), acrylics, vinyl resins, and carboxylic-containing copolymers
chemical reaction hardening	thermosetting polymers such as cyanoacrylates, epoxy, phenolics, polyester, formaldehyde, and polyimides
pressure sensitive	natural rubber, styrene–butadiene rubber, butadiene-acrylonitrile rubber, acrylics, and atactic polypropylene

In addition to the main polymer, other components can be found in commercial formulations. Thermosetting adhesives may contain hardeners that promote the curing reaction acting as a catalyst or cross-linker.^17^ Solvents are needed to reduce the viscosity of synthetic resins and elastomers.^18^ Fillers improve the mechanical or resistance properties.^19^ Other additives such as plasticizers, inhibitors, tackifiers, thickeners, and antioxidants are sometimes included to enhance the properties and provide broader functionality.

Synthetic adhesives often exhibit hazardous properties determined by the following factors: corrosivity, reactivity, toxicity, and ecotoxicity. Most current adhesives are toxic due to the presence of volatile organic compounds (VOCs).^8^ Exposure to these components produces a health hazard, and inadequate recovery and reprocessing technologies limit their inclusion in a zero-waste industry system.^20^ Specialty or structural adhesives represent a challenging subject given their adhesion strength, which is designed to withstand adverse mechanical, thermal, or chemical conditions.^21^

Increasing amounts of solid waste contribute to the overcapacity of landfills and the contamination of soil and water. When unrecycled or defective items are disposed of as general waste, they contribute to these problems, hence the need for a new generation of adhesives that would allow the dismantling of products at the end of their lives and the recovery of waste suitable for recycling, reusing, or repurposing.

## Adhesives for a Zero-Waste Industry

The role of adhesives in a zero-waste industry is often underestimated as they represent a secondary component of the end product. However, industrial expansion and the emergence of new products and applications are leading to increased demand for adhesives.^19,22^ The need for a new generation of zero-waste adhesives comes from an understanding of the impact that chemical and material waste has upon human and environmental health.

The key environmental issues linked to the use of adhesives are related to the presence of VOCs, the use of nonrenewable petrol derivatives in the contamination of waste intended for recycling, and problems due to leaching out once landfilled.^23^ However, the main concern is not only the adhesive itself but also how its presence limits the reincorporation of the materials it bonds into the zero-waste system.

For an adhesive to reduce its environmental impact and therefore ease the inclusion of adhesives and adhered components into a zero-waste industry, three main complementary strategies can be implemented:

1. development of adhesives based on biosources;
2. development of dismantlable or reversible adhesives; and
3. development of recyclable adhesives.

Adhesives that display one or more of these properties are needed to facilitate a zero-waste industry, though recyclable adhesives have not been generally studied and reported. The following sections explore the most recent advances and trends in the first two strategies enumerated above and how the chemical identity can deliver novel adhesives from the perspective of a zero-waste industry.

## Development of Adhesives Based on Biosources

The selection of raw materials is fundamental to producing adhesives that reduce hazardous emissions and waste. Adhesives based on biosources exhibit distinctive characteristics from synthetic adhesives mainly because of their versatility.^24^ They are capable of binding to diverse substrates regardless of their roughness, surface fouling, and in some cases humidity. Their production is usually a one-pot process at room temperature and atmospheric pressure.^10^

Two categories of biobased adhesives are established: natural adhesives, formulated from vegetable sources, and biological adhesives, which comprise the secretions of organisms with adhesive properties. A third category is sometimes defined to be biomimetic surfaces, where the topology resembles those observed in nature (e.g., gecko feet and insect wings).^25−27^ As their synthesis requires lithographic, etching, or demolding processes and their behavior is more closely related to their mechanics, biomimetic adhesive surfaces will not be considered in this Feature Article.

Natural adhesives were the most popular adhesives until synthetic ones became available.^16^ Though their use has decreased because of the development of superior and more reliable adhesives, there has been a renewed interest due to their inherent sustainability. Natural adhesives are widely available, nontoxic, biodegradable, generally low cost, and mostly carbon-neutral.^10^ These natural adhesives include starch, cellulose, plant oils, and natural rubber.

### Starch

After cellulose, starch is the most abundant natural organic compound. It is usually found in roots, tubers, leaves, fruits, and many others with corn, potato, and wheat being the main sources.^28^ Starch is composed of two main polysaccharides: amylose and amylopectin.^29^ While films from amylose are strong and tough, those from amylopectin are soft and weak. It is the botanical source of starch (i.e., from where it is extracted) that determines its physicochemical properties.^30^ However, prior to being used to produce adhesives, starch needs to be modified to improve its shear strength, thermal and humidity stability, degree of retrogradation, and bonding strength.^28^

Starch oxidation,^31,32^ grafting,^31,33^ cross-linking,^31,34^ and esterification^31,35^ are the main routes to improving native starch properties. Among these, graft copolymerization is one of the preferred methods as it allows a degree of control over the resulting properties of the starch derivative.^28^ A cassava starch-based wood adhesive grafted with itaconic acid (IA) was developed.^36^ Native starch possesses hydroxyl groups, which are responsible for its low water resistance. Through graft copolymerization it is possible to reduce the quantity of these groups and improve the water resistance. Itaconic acid, an unsaturated binary acid, is obtained from agricultural products instead of petrochemical sources, which is a problem with acrylic or methacrylic acids. Carboxylic side groups in IA can form hydrogen bonds with hydroxyl groups in starch, hence decreasing water absorption.

Optimal mechanical properties in the dry state were observed^36^ in adhesives containing 5 wt % IA (starch basis) with a single-lap shear strength of 15.4 MPa, 43.6% higher than for unmodified starch. Adhesives with 7.5 wt % IA achieved 5.2 MPa under wet conditions, 2.2 times higher than for pristine starch. Considering that the wet shear strength of 5 wt % samples was only 12% lower than that of 7.5 wt % samples and the cost of IA, starch-based adhesives with 5 wt % IA are better candidates for an industrial setting. These shear strengths are comparable to curing hot melt adhesives and some cyanoacrylates.^37^

In another study,^38^ urea-oxidized corn starch (oxidized with potassium permanganate) was prepared through polycondensation and modified with nano-TiO_2_ (titanium dioxide nanoparticles) to improve the adhesion properties and stability of the adhesive. The maximum dry shear strength achieved by the nanocomposite adhesive was 2.4 MPa at 1.5 wt % nano-TiO_2_, 5.0 wt % oxidizer, and 50 wt % urea, all with respect to dry starch. This was 16.9% higher than in absence of nano-TiO_2_. The wet shear strength was reported to be 0.97 MPa. Conventional urea-formaldehyde wood adhesives possess a dry shear strength of ≥1.9 MPa and a cost of between 1 and 3 dollars/kg. The reported cost for this adhesive is 0.5 dollars/kg.^38^ Not only does this adhesive compete with current synthetic wood adhesives but it is also renewable and environmentally friendly.

Adhesives with enhanced shear strengths can also be efficiently prepared by oxidizing starch with hydrogen peroxide (H_2_O_2_) and using a silane coupling agent as a cross-linker and an olefin monomer.^28,39^ Other methods include adding surfactants along co-monomers and fillers (e.g., clays).^28,40,41^ The potential of starch as an adhesive relies on its versatility to be modified and therefore enhance its adhesive and wear resistance properties. In addition, its moderate cost makes it a suitable candidate as a raw material in industrial applications, mainly as a wood adhesive replacing urea formaldehyde, melamine-urea formaldehyde, and other VOCs.^42^

### Cellulose

Cellulose is a polysaccharide constituted of β-linked d-glucose units that form plant cell walls.^43^ It is the most abundant biopolymer and has a wide range of applications in the textile and paper industries.^44^ The main limitation of cellulose as an adhesive is its solubility. Cellulose does not dissolve in conventional solvents, which has impeded the formulation of cellulose-based adhesives.^45^ The main research has focused on developing greener solvents,^46^ but related studies have found that an alkaline cellulose solution can be used to prepare polymeric materials and nanocomposites.^47^

It has been suggested that the adhesive properties of alkaline cellulose solutions have not been studied due to the stigma surrounding cellulose and their insolubility. To study this, the adhesion of alkali cellulose solution-bonded filter paper and sulfite writing paper was tested.^44^ A 5 wt % cellulose solution was prepared in a 7 wt % NaOH aqueous solution at 0 °C, followed by freezing at −20 °C for 1 h and defrosting at room temperature. Single lap joint, butt joint, and impregnated paper samples were prepared and tested through uniaxial tensile tests.

**Figure 1:**
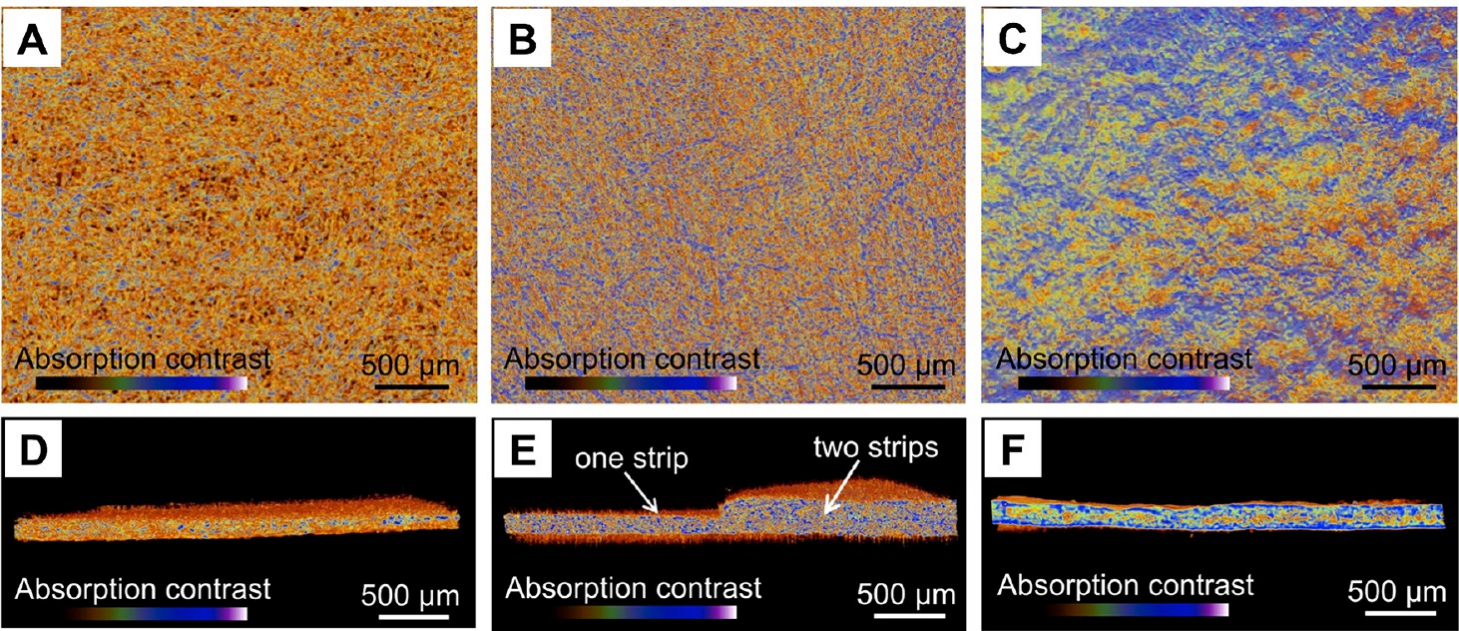
X-ray microtomography of top and cross sections of (A, D) pristine filter paper, (B, E) a single lap joint, and (C, F) impregnated filter paper. Reprinted with permission from Ferreira et al.^44^ Copyright 2015 American Chemical Society.

For filter paper, it was found that the tensile stress in bonded samples was comparable to that of pristine paper at ca. 12 MPa, while for impregnated filter paper it increased to 18 MPa. Lap joint sulfite paper achieved a maximum stress of 60 MPa, comparable to that of a control sample and 5 times that of the butt joint. Overall, bonded areas improved their resistance as fracture never occurred at the adhesive joint. Microscopy and microtomography imaging showed that cellulose penetrated the paper (Figure 1A–F), which is as fundamental to adhesion as other interactions, be they electrostatic, van der Waals, hydrogen bonds, hydrophobic, or chain interdiffusion.^44^ Such interactions are essential not only for this study but also for all adhesion processes.

As for starch, the hydroxyl group of cellulose can be modified to improve its solubility and decrease its crystallinity, which limits its use in adhesive formulations.^43^ Microcrystalline cellulose (MCC) was oxidized selectively using sodium periodate to form dialdehyde cellulose (DAC).^48^ DAC was then further processed with three different polyamines (PA_4N_, PA_5N_, and PA_6N_) to form a covalently cross-linked hyperbranched polymer network. The resulting adhesives were water-resistant with enhanced lap shear strengths. Optimal samples containing PA_4N_ showed dry strengths of 3.3 MPa, 123.8% higher than that of pristine DAC, and 2.27 MPa when redried after 3 h of immersion in water at 63 °C. Such characteristics make these adhesives more robust and capable of coping with more extreme environments and applications (e.g., water erosion, underwater, and above-room-temperature environments). Conventional adhesive sealants and hot melts display similar shear strengths, 2.0–5.5 and 1.7–4.8 MPa, respectively, under dry conditions.^49^

Cellulose has also been used in its nanocrystalline form (CNC) to reinforce and stabilize adhesive formulations as it produces colloidal aqueous dispersions.^50^ To demonstrate improved emulsion pressure-sensitive adhesives, two types of CNCs, CNC101 and CNC103, were used to prepare butyl acrylate, vinyl acetate, and acrylic acid copolymer emulsion nanocomposites.^51^ CNC101 consisted of the spray-dried powder form of the sulfate sodium salt of unmodified CNCs, and CNC103 was a proprietary Jeffamine-modified CNC. Compared to CNC101, CNC103 possessed a smaller zeta potential, a lower surface tension, and a reduced hydrophilic nature, leading CNC103 to surround the polymer latex particle more effectively than CNC101.

Overall, the tack, peel strength, and shear strength increased with the addition of CNC, compared to pristine copolymer formulation.^51^ Maximum values of tack (PSTC16 standard) were achieved at ∼450 N m^–1^ for adhesives with CNC101 at 0.75 phm (parts per hundred monomer) and CNC103 at 1.00 phm, ∼2.2 times the tack of pristine latex. Notably higher values of the peel strength (PSTC101 standard) were displayed for CNC103 than for CNC101. For adhesive samples with CNC101 at 0.75 phm content, the peel strength was ∼120 N m^–1^, while for samples containing CNC103 at 1.25 phm, measurements reached 200 N/m. This behavior is caused by CNC101 nanoparticles hampering the deformation of polymer chains, hence decreasing their energy dissipation. Control samples showed a peel strength of just around 10 N m^–1^. Finally, the shear strength (PSTC107A standard) greatly increased with the addition of CNC101 at 0.75 phm content, reaching 110 h. For CNC103 at 1.0 phm content, the shear strength showed a limited increase compared to that for pristine latex (20 h), below 40 h. The shear strength is influenced by cross-linking and entanglements, which appeared to be favored in the presence of CNC101. The overall adhesive behavior is related to the stiff structure of CNC, the formation of CNC aggregates, the interfacial forces and polymer chain mobility, and the stretch and entanglements.

CNCs represent a useful resource in the formulation of waterborne polymer adhesives as has been reported previously.^52,53^ Though polymers in these systems may not be ecofriendly, using water instead of an organic solvent represents an advantage. There has been a special interest in water-based emulsion formulations because this procedure is already being carried out at an industrial level to produce paints, coatings, and some adhesive formulations. Emulsion polymerization offers the additional advantages of allowing the tailoring of viscosity, providing high reactions rates, and being environmentally friendly.^54,55^

The role of CNCs as stabilizers for emulsion polymerization has also been studied. One report^55^ showed the synthesis of CNC-stabilized emulsions of vinyl acetate (VAc) polymerized with and without poly(ethylene glycol) methacrylate (PEGMA) and their application as a waterborne adhesive. Unfilled PVAc adhesive achieved a shear strength of 4.3 MPa, while PVAc with 8 wt % CNC increased it about 200%. The inclusion of PEGMA further enhanced the shear strength by more than 230%. This occurred because PEGMA favors the accumulation of CNC on the PVAc particles, costabilizing the dispersion. Research on fully environmentally friendly water-based emulsion adhesives is still ongoing. Overall, systems of this kind should be made from green stabilizers and polymers, minimizing adhesive loss.^42^

In another study,^56^ biodegradable adhesives prepared from poly(glycerol succinate) (PGSu), poly(glycerol maleate) (PGM), or poly(glycerol maleate-*co*-succinate) (PGMSu) and reinforced with 5, 10, and 20 wt % CNC were analyzed. The shear strength was tested on Angelim wood and compared to a PVAc commercial adhesive. While pristine PGSu and PGM displayed shear strength similar to that of PVAc (1.59 MPa), the PGMSu copolymer doubled this value. Furthermore, shear strength increased upon the inclusion of CNC with a maximum strength of 4.14 MPa for PGMSu 10% CNC. CNC enhanced not only the adhesive behavior but also the thermal properties and degradation resistance.

The use of cellulose in new adhesive formulations has been motivated by its natural abundance (annual rate of 10^11^–10^12^ t).^57^ It can be used in different polymer systems and can perform varied roles from the main constituent to a stabilizer and filler. This versatility makes cellulose a material worthy of further research. However, it should be considered that although CNCs provide greater functionality than conventional cellulose, their high water content and production cost remain limitations for their widespread use as sustainable solutions.^58^

### Plant Oils

Plant oils have captured the interest in these applications because of their availability, biodegradability, low cost, and low toxicity.^59^ Their reactivity enables their functionality to be tailored to practical applications, such as adhesives. In one example,^60^ pressure-sensitive adhesives (PSAs) were prepared from two biosources: soybean oil, which is a plant oil, and lactic acid oligomers (OLA), which are polysaccharide derivatives. Lactic acid oligomers were synthesized without catalysts or solvents and then UV polymerized with epoxidized soybean oil (ESO).

Optimized copolymers of short oligo-lactic acid chains (260 ± 0.5 g mol^–1^) with molar ratios of OLA to ESO of 2.5–3.5 displayed a peel adhesion strength of up to 3.8 N cm^–1^, tack adhesion of 8.0 N cm^–1^, and shear adhesion resistance of 30 000 min with a test mass of 1000 g.^60^ The molecular structure was flexible enough to display adequate adhesion with glass transition temperature, *T*_g_, between −20 and 0 °C. In addition, the polar groups (−COOH and −OH) promote the formation of noncovalent substrate bonding. These copolymers represent a fully biobased adhesive alternative for general purpose tapes and labels as PSAs are usually prepared from acrylics, silicones, polybutadienes, and their copolymers.^61^ Replacing these petrochemicals with renewable resources is not only economically attractive but also environmentally responsible.

Among conventional adhesives, polyurethanes (PUs) are widely used because of their adhesion strength and resistance to weathering. PUs are prepared from a soft segment of polyols and a hard segment from diisocyanate.^62^ Polyols are obtained from petrochemical sources in various grades, depending on the intended application. However, new sources and biobased polyols are being studied.^63^ Vegetable oils are an alternative, but they generally display low viscosity, rendering them inappropriate as they generate low thermal and mechanical stability.^63,64^

In one example, a biobased polyol was prepared from ESO and then modified with tetraethyl orthosilicate as a means to improve viscosity and stability.^63^ This polyol was reacted with polymeric 4,4′-diphenylmethane diisocyanate along with three additives: zinc oxide nanoparticles, triethylene glycol (TEG) as a chain extender, and dibutyltin dilaurate (DBTDL) as a catalyst. The Taguchi method was used to optimize the additive content, obtaining for the ZnO nanoparticles 0.1%, TEG = 1%, and DBTDL = 0% by mass. The lap shear strength reached 6.48 MPa, close to the predicted value of 7.17 MPa. Moreover, the modulus increased from 0.27 GPa for the control adhesive to 0.37 GPa for the optimum formulation. The optimum adhesive was thermally stable up to 220 °C. It was found that the inclusion of ZnO nanoparticles was the predominant factor in the adhesive and thermal properties of the material. The shear strength is comparable to those of commercial PU adhesives, which are between 2.8 and 7.0 MPa.^49^

The introduction of green priorities in the chemical industry has led to the search for natural derivatives that can simultaneously address environmental concerns and performance requirements. For instance, the viscoelastic tuning of pressure-sensitive adhesives is usually achieved with the inclusion of tackifiers (to improve adhesion and increase *T*_g_), plasticizers (to decrease hardness and *T*_g_), and stabilizers.^65−67^ Styrenic block copolymers present a common example.^68^ These are typically ABA copolymers of hard A blocks with high *T*_g_ and soft and rubbery B segments.^69^

**Figure 2:**
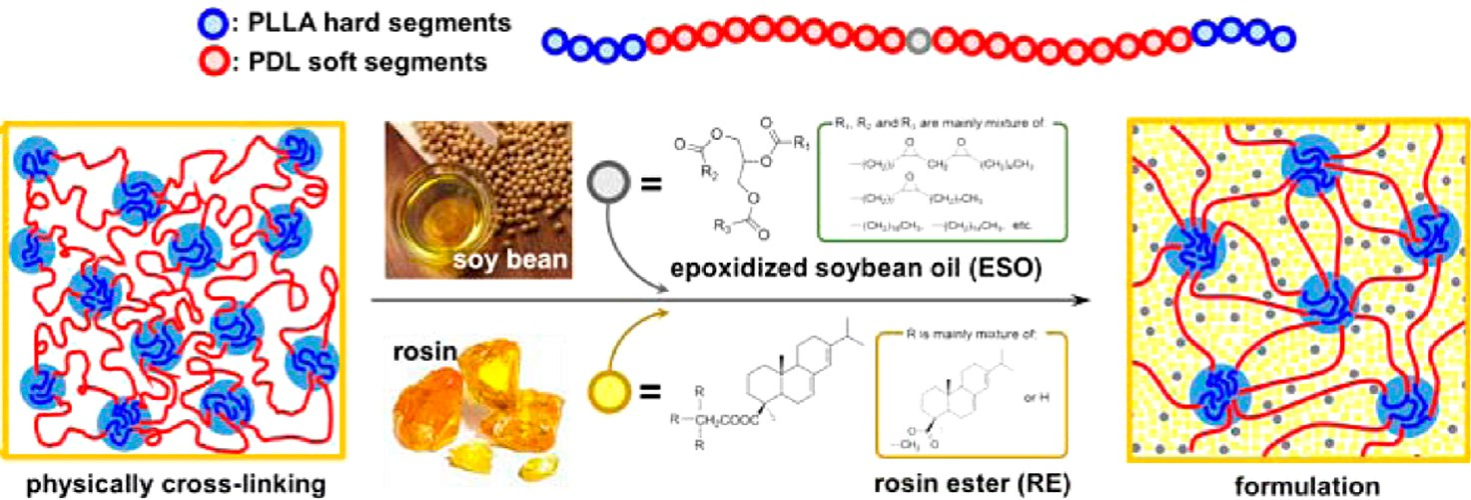
Schematic representation of the synthesis of renewable PSA formulation. Reprinted with permission from Lee et al.^69^ Copyright 2015 American Chemical Society.

To address the need for natural derivatives, a PSA based on the poly(l-lactide)-*block*-poly(ε-decalactone)-*block*-poly(l-lactide) (PLLA–PDL–PLLA) thermoplastic polyester elastomer was prepared through ring-opening transesterification polymerization, using rosin ester (RE) as a tackifier and ESO as a plasticizer (Figure 2).^69^ PSAs containing 35 wt % elastomer, 50 wt % RE, and 15 wt % ESO achieved peel adhesion values of up to 2.6 N cm^–1^ without adhesive residue, tack forces of between 1.0 and 3.0 N, and a shear strength of ca. 23 000 min. These values are comparable to those of commercial PSA tapes. The weakness of this kind of synthesis is the need for a metal catalyst, tin(II) ethylhexanoate in this case.

Epoxidized soybean oil and other modified triglycerides have attracted interest not only because they are produced from natural, renewable materials but also because they are capable of producing adhesives with thermal stability, transparency, chemical resistance, and peel strength comparable to those of conventional PSAs.^69,70^ By carefully selecting the plant oil and its functionalization, one can produce initial monomers such as fatty acids, polyols, polyacids, and epoxides that can form linear or cross-linked polymers.^69^

Contrary to other biomolecules (e.g., polysaccharides and proteins), plant oils have an advantage by introducing hydrophobicity due to the alkyl chain of fatty acids.^71^ Other advantages include their role as plasticizers,^72^ as segments in polyurethanes,^63^ and as epoxy-cross-linkers after epoxidation.^73^ Still, the control of these structures and especially the stoichiometric control of functional groups are limited due to the varying compositions of triglycerides that depend on the crop, season, and growth conditions.^69^

### Natural Rubber

Another plant-extracted material is natural rubber, a biopolymer of *cis*-1,4-polyisoprene with a mixture of proteins, carbohydrates, and lipids in an aqueous phase, from the tree *Hevea braziliensis*.^74^ It is an elastomeric biopolymer showing good flexibility, strength, and tack. However, it is difficult to bond with high-surface-energy materials requiring modification through epoxidation,^75^ chlorination,^76^ or grafting.^77^ Natural rubber grafted by methyl methacrylate (MMA) at different solid contents has also been prepared.^78^ The lap shear strength was determined, and the best value was 1025 kPa at a total solid content of 57%, which did not vary with the MMA content. However, it does rely on the storage time as it was found that the lap shear strength decreased progressively to just over 700 kPa at week 4. Additionally, the contact angle decreased from 95° for natural rubber to 65° for the modified rubber, making the adhesive more compatible with materials such as wood and other synthetic rubbers. The reported shear strength was within the range of that reported for foam adhesive tapes (0.3–3.0 MPa).^49^

As discussed above, starch is a biopolymer that has been used in the production of adhesives. The hydrophilicity of starch is incompatible with the hydrophobic nature of natural rubber, which would weaken their resulting composite.^79^ To overcome this limitation, a latex compounding method was demonstrated to allow the dispersion of starch in a natural rubber matrix.^80^ It consisted of gelatinizing starch at 90 °C, which decreases the particle size and enhances dispersion. Chemical^81^ and physical^82^ modifications of starch are also routes to achieving compatibility with natural rubber.

The effect of nanosilicates incorporated into natural rubber-based adhesives has also been studied.^83^ The peel strength for natural leather joints was reported. Two nanosilicates designated as NS1 and NS2 were compared to kaolin clay, and it was found that their inclusion improved the peel strength by 127% at 2 parts per hundred rubber (phr) content of NS1 and by 85% at 1 phr of NS2. In contrast, the addition of kaolin clay decreased the peel strength compared to that of pristine natural rubber. After thermal aging for 100 h at 70 °C, the peel strength increased by 165 and 68% for the same NS1 and NS2 contents. These results were explained by electron microscopy, which showed a better dispersion and exfoliation of NS1 compared to that of NS2. The inclusion of these nanosilicates also improved the thermal stability and wettability of the adhesive film due to the enhanced barrier properties.

Natural rubber is one of the most promising natural adhesives as its processing has been extensively studied as for the production of household and engineering goods, especially after vulcanization became widespread.^84^ Its modification enhances its adhesive properties, making this natural material of interest for new sustainable adhesive applications.

### Biological Adhesives

Plants are not the only source of biobased adhesives. The shear adhesive properties of the bacterial polysaccharide FucoPol were studied.^85^ FucoPol was produced from *Enterobacter A47* in glycerol and extracted. The adhesive was prepared by heating a 3 wt % FucoPol solution at 50–60 °C for 6 h to obtain a concentrated solution of about 7.6 wt %. Sodium azide at 10 ppm was added to prevent microbial growth. The adhesion strength was determined for balsa wood, glass, cellulose acetate film, and corrugated cardboard. For wood, the maximum strength provided by the equipment (742.2 ± 9.8 kPa) could not detach the specimens. For cardboard, glass, and cellulose acetate, the maximum shear strengths were 416, 115.1, and 153.7 kPa, respectively. These values are similar or even superior to those of commercial UHU glues. Though FucoPol is susceptible to humidity, it can be useful for book manufacturing, pressure-sensitive tapes, labeling, or medical adhesives.

Substances obtained from bacteria or fungus have the potential of being developed into adhesives that are nonhazardous and environmentally friendly. Proteins and polysaccharides from biofilms provide adhesive interactions and cohesive strength^86^ as they form hydrogen bonds. This, however, limits their applications as they tend to form gels when hydrated.^87^ Some of these polysaccharides are already being used in industry as thickening and gelling agents, for example, xanthan gum (*Xanthomonas campestris*), gellan (*Sphingomonas paucimobilis*), and dextran (*Leuconostoc mesenteroides*) or scleroglucan and pullulan from a fungal origin.^88^

Fungal mycelium contains proteins and glycoproteins that modify the surface energy of the substrate acting as an adhesive.^89^ Natural mycelium from different types of mushroom substrates has been used as a bioadhesive to produce board material.^90^ Spent mushroom substrates (mushroom compost) were grown from sawdust, food waste, or diaper waste. These were then dried, blended, and compressed in a mold at 160 °C and 10 mPa for 20 min to form boards. It was found that boards showed internal bond strengths in the range of 1.34 to 2.51 mPa. These values are greater than those of industrial particleboard (0.31–1.17 mPa). Bioboards such as these could be used to manufacture furniture as their mechanical properties exceed U.S. and China standards.

Gastropod mollusks secret adhesive mucus capable of adhering under wet and dry conditions. For instance, the adhesive strength of the mucus of snail *Macrochlamys indica* has been characterized under different conditions.^91^ The adhesion strength was significantly higher in the presence of alkaline buffers (6.5 kPa on average) than under acidic conditions (2.5 kPa), which can be considered to be extreme conditions. These measurements are in accordance with the intrinsic alkaline pH of the mucus. The difference in strength is related to the superior water content in the mucus and its ability to absorb additional water at alkaline pH. Such behavior could be useful in the development of biobased adhesives that display tailored strength in wet environments.

Biobased compounds are the primary alternative in the development of adhesives for a zero-waste industry because they are renewable resources with a low carbon footprint, they are biodegradable, and they are usually nontoxic. These characteristics make them compatible with more stringent health and environment policies.

As reviewed, one can produce adhesives directly from biopolymers and in some cases from monomers obtained from natural sources. A summary of the main considerations for each of the sources described can be found in Table 2. The variety of biobased molecules provide a wide library of raw materials that can be modified or used as-received, further increasing the functionality of adhesives and providing versatile bond formation, various degradation pathways, and tunable properties.

**Table 2:** Relevant Bioadhesive Sources

bioadhesive source	remarks
starch	■ needs to be modified (e.g., oxidation, grafting, cross-linking, or esterification) to improve shear strength, thermal and humidity stability, degree of retrogradation, and bonding strength
cellulose	■ poor solubility in conventional solvents
	■ can be used as fibers in its microcrystalline or nanocrystalline form or as the main constituent, stabilizer, or filler
plant oils	■ provide thermal stability, chemical resistance, and peel strength
	■ introduce hydrophobicity and act as plasticizers or cross-linkers
	■ can be used to produce initial monomers that are fatty acids, polyols, polyacids, epoxides, etc.
natural rubber	■ provides flexibility, shear strength, and tack
	■ difficult to bond with high-surface-energy materials but it can be modified to improve this (e.g., epoxidation, chlorination, or grafting)
biological adhesives	■ difficult to obtain in large quantities
	■ generally poor mechanical properties and susceptible to humidity

The current challenges for the use of biobased materials in the adhesive industry are their susceptibility to hydrolytic degradation, the limited reproducibility among batches due to crop differences, and the competition with food production. Besides, these adhesives still need to be removed through dissolution or mechanically. Despite these limitations, the use of biobased adhesives increases the possibilities for future zero-waste industries. Although biopolymers have been used before and are now undergoing a resurgence, bioderived monomers are showing greater promise because they allow superior control and versatility in adhesive production.

## Development of Dismantlable or Reversible Adhesives

A term often used in environmental issues is “3R”, which stands for “reduce”, “reuse”, and “recycle”. Though this concept can be considered simplistic, it is relevant for a zero-waste industry to succeed.^23^ The basic strategy to reduce the environmental impact of adhesives is the optimization of the amount of adhesive used, which could be interpreted as “reduce” in a similar way as decreasing the consumption of manufactured goods would be, especially single-use products. Furthermore, “reusing” and “recycling” would imply the separation of adhesively bonded materials. To reuse defective products or recycle bonded materials, dismantlable or reversible adhesives are being developed.

Dismantlable adhesives are those that can be separated on demand through the application of an external stimuli:^23^ a change in temperature,^92,93^ exposure to radiation,^94,95^ an electrical current,^96,97^ or a change in pH,^98,99^ among others. Being able to detach bonded materials can be useful for reducing waste associated with defective products that cannot be repaired due to failed parts bonded adhesively or to bonded materials that cannot be reintegrated into the production flow without being first separated.^7,23^

Dismantlable adhesives are usually classified into three categories:

1. thermoplastics and adhesives containing expansion agents;
2. electrochemically dismantlable adhesives; and
3. chemically dismantlable adhesives.

Dismantlable adhesive mechanisms rely on physical and chemical interactions and changes. Detachment can be promoted by heat inducing a physical change (chain mobility), decomposition reactions, redox reactions, phase changes, electrostatic interactions, cross-linking, or bond scission among other processes.^100^ In the following sections, the different mechanisms for on-demand debonding are identified and exemplified.

## Thermoplastics or Adhesives Containing Expansion Agents

Thermoplastic adhesives are the simplest dismantlable adhesives as they are softened by heating at a specific temperature. These are typically used for high assembly speed applications, and they have the advantages of reducing environmental hazards associated with solvents, solvent vapors, and waste during application.^20,101^ In addition, sources of heating are suitable for automated assembly and include direct methods such as hot air and heated tools and indirect methods such as ultrasound and lasers.^23,102^

Hot-melt adhesives allow bonding substrates made of different materials and have become of particular interest in the electronics, packaging, construction, and automotive industries. For instance, in the production of integrated circuits a dismantlable wax or adhesive is used to fix silicon ingots to be cut into wafers.^23^

Nowadays, the “all-over method” is used by construction companies as it allows bonding wall boards on beams. It consists of a low-tack hot-melt tape embedded with an aluminum alloy layer. Once the board is positioned, it is heated by induction until the tape softens and joins the beam. The same process is followed to separate them.^103^ In the automotive industry, adhesives have been used instead of fasteners and welds to reduce weight and therefore fuel consumption. Hot melts are currently used for interior and exterior parts such as panels, instrument gauge springs, pipes for air conditioning, bumpers, plates, lamps, and others.^104^

The main limitations for the use of thermoplastics are the limited thermal stability which makes them unsuitable for certain applications, the loss of strength as the temperature rises even below the melting temperature (i.e., causing creep), and their sensitivity to moisture or other environmental factors.^100^ In addition, some substrates may be susceptible to heat. To overcome these challenges, the combination of thermosetting and thermoplastic polymers to produce dismantlable adhesives has been researched.^105^

A route to further increase the environmental friendliness (e.g., ease of residue elimination) of thermoplastics is to produce them from biopolymers and biodegradable materials. Polymer blends based on thermoplastic biopolymers (e.g., thermoplastic starch) have been developed,^106^ along with biopolymer composites,^107^ thermoplastic polyurethanes with biodegradable segments,^108^ and other multiblock thermoplastic materials based on natural monomers.^109^ However, these would display some of the limitations related to bioadhesives.

Other well-studied dismantlable adhesives include those based on thermally expandable microcapsules (TEMs). TEMs are blowing agents that expand when heated, producing an internal stress within the adhesive matrix.^101^ The expanding force produces deformation and consequently failure in the adhesive–substrate interface. The first reports on this type of adhesive consisted of resins with plastic shells containing liquid hydrocarbon. For instance, a bisphenol A epoxy resin adhesive matrix containing TEMs made of a poly(vinyl chloride) shell and a liquid hydrocarbon core was reported.^110^ To test the dismantlement, aluminum alloy plates (gridblast and gridpaper finishing) were bonded with the adhesive and then immersed in boiling water or exposed to hot air at 100 °C. It was found that at 50 wt % TEM content the adhesive expanded by a factor of 4. Lap shear tests were performed, showing a maximum value of 12.3 MPa for the gridblast finishing condition and 7 MPa for gridpaper finishing. Shear strengths compete with those of commercial structural adhesives (≥10 MPa).^37,49^

Conventional TEMs are usually made of a thermoplastic polymer shell (e.g., poly(acrylonitrile), poly(vinylidene chloride), polyolefins, or poly(methyl methacrylate)) and a hydrocarbon core with a low boiling point such as petroleum ether, hexane, or butane.^111^ The surface of TEMs can also be modified to improve their compatibility with adhesion promoters, which not only enhance the overall mechanical properties but also can improve their thermal response.^112^ Besides their obvious downside, hydrocarbons are liquids at ambient temperature, and due to their low boiling point, they vaporize easily and must be processed at high pressure. Using other blowing agents would be conceived as a superior alternative to VOCs.

Previously, TEM-encapsulated ethanol had been reported as a substitute for hydrocarbons as blowing agents.^113^ More recently, environmentally friendly TEMs have been prepared by polymerizing a shell of acrylonitrile (AN), MMA, and methacrylate (MA) and using water as the blowing agent.^114^ The optimized TEMs were prepared with an AN/MMA/MA monomer ratio of 1:2:2 and 1,4-butanediol dimethacrylate at 0.2 wt % with respect to total monomer mass as a cross-linker. These TEMs contained 35.2 wt % water and had an average diameter of 35.9 μm. Their expansion was evaluated in a melamine resin and compared to *n*-octane and TEM-encapsulated *n*-octane. It was found that the expansion ratio of TEM-encapsulated water and *n*-octane was 4, while materials foamed using *n*-octane had an expansion ratio of 5.

**Figure 3:**
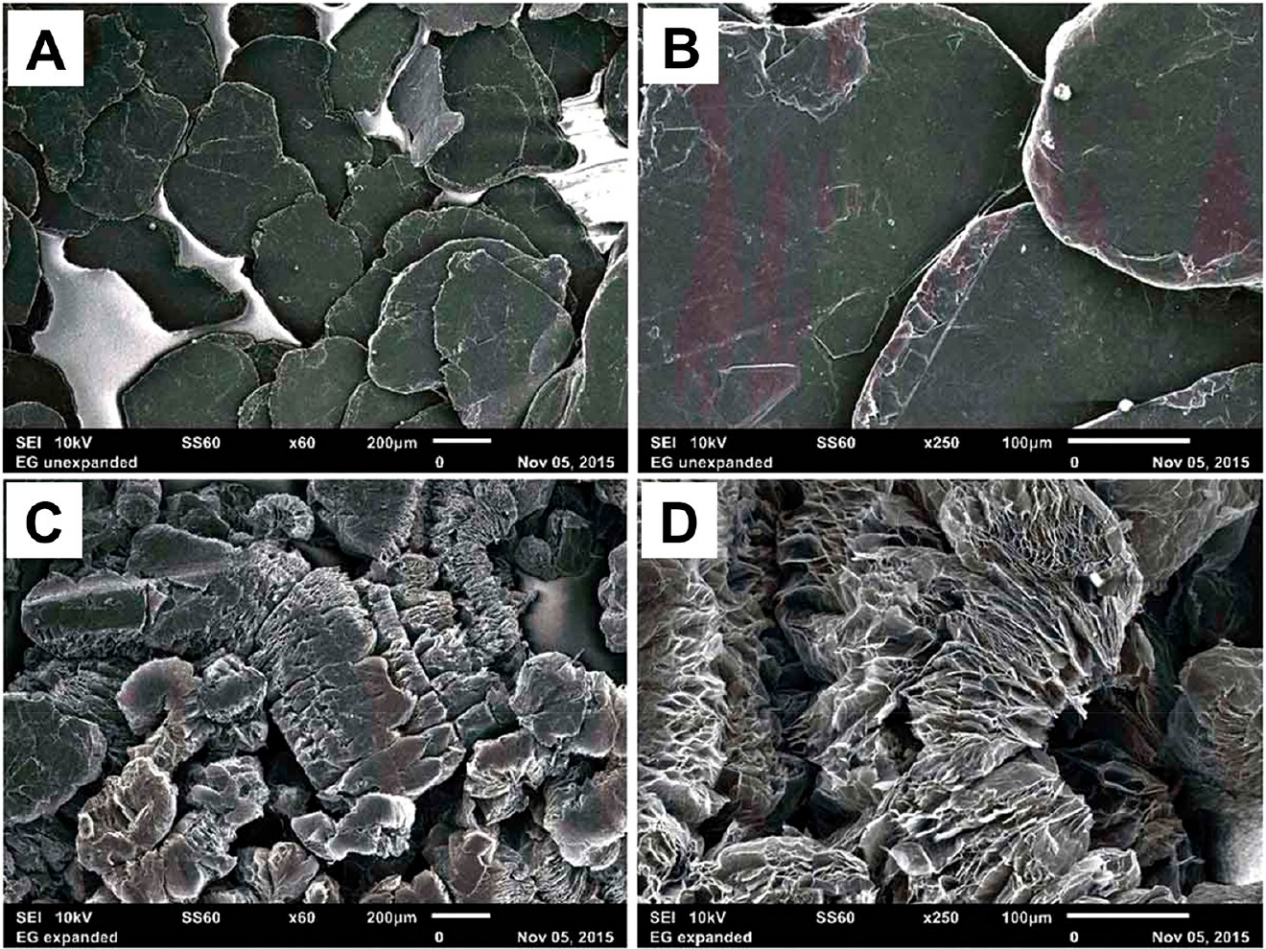
Graphite flakes. (A, B) Natural flaky lamellar structure at 60× and 250× magnifications. (C, D) After heat-triggered expansion/exfoliation at 60× and 250× magnifications. Reprinted with permission from Pausen et al.^115^ Copyright 2016 Taylor & Francis.

TEMs have been replaced with other species, for instance, expandable graphite (EG) which was incorporated into an epoxy and PU adhesive.^115^ EG is a form of graphite containing intercalated compounds (i.e., sulfuric and nitric acid anions) that decompose into gas and water when heated, causing flakes to expand (Figure 3). Short- and long-term lap shear strengths were evaluated in aluminum alloy substrates. It was found that the optimal EG content was 5 wt % for PU and 3 wt % for epoxy resin. At these concentrations, the tensile shear strengths were 16.9 and 26.6 MPa with complete separation of the joints at 235 °C. The lap shear strength was also evaluated for epoxy samples after 90 days of immersion in water at 55 °C. Tests showed a loss of 22% of the initial strength for the 3 wt % EG samples.

Thermal volumetric expansion of fillers as a means to induce dismantlement is possible for different systems containing dilated vermiculite, pearlite, mica, wermlandite, thanmasite, and hydrotalicite in the range from 25 to 70 wt % of the curable resin and with a heat expansion temperature of between 250 and 500 °C.^116^ This approach has the advantage of decreasing the use of petrochemical derivatives compared to conventional TEMs.

Adhesive thermoplastics and those containing expansion agents do not require complex chemical synthesis, hence their translation into an industrial setting would be largely straightforward. Research on this kind of adhesive technology should focus on minimizing or removing the use of petrochemicals as well as optimizing the temperature at which debonding occurs according to the application requirements.

## Electrochemically Dismantlable Adhesives

Electroadhesives display a reversible behavior when an electric potential is generated in the interface of adhesion. Detachment can occur through faradaic reactions, phase separation, ion mobility at the substrate–adhesive interface, gas emission, and mechanical stresses, among other mechanisms that are still being identified and studied.^117^ These systems provide precise control of debonding, rapid inducement, and ease of operation, which makes them particularly useful in haptics and robotics, automotive, and aerospace applications.^100^

The first electrically assisted debonding system was developed to fulfill the requirements for aerospace applications from the U.S. Air Force. ElectRelease by EIC Laboratories was developed for this purpose.^101^ This adhesive is an amine-cured epoxy between two metal plates that can be detached at the positive interface upon the application of a voltage of 10–50 V (current flow of 1–5 mA cm^–2^).

The main limitation of electrically triggered debonding is that substrates must be conductive, which makes it unsuitable for a wide range of applications. Electrodes include metals (e.g., aluminum, steel, copper, and titanium) and, in the case of the anode, not only metals but conductive polymer composites or other similar materials.^23,118,119^ Adhesives of this type often include additives (e.g., salts) that provide ionic conductivity, so the adhesive behaves as an electrolyte between two substrates acting as electrodes.^117^

Electroadhesives based on faradaic reactions and nonfaradaic effects are still being developed. These include capacitive ion storage, ion mobility, surface charges, water electrolysis, and redox reactions.^117^ The variety of mechanisms that allow electrical reversibility has pushed forward the study of chemical compositions that can be used in these adhesive systems.

**Figure 4:**
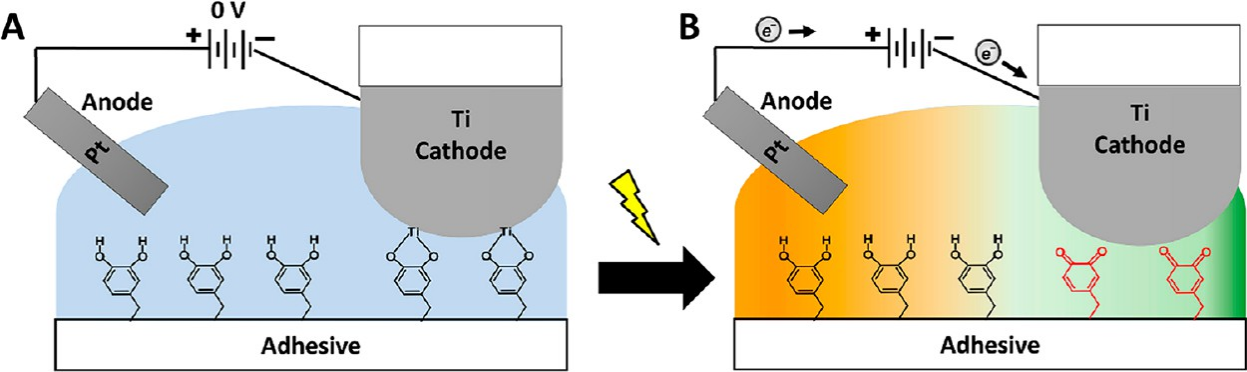
(A) Catechol adheres to the Ti cathode at 0 V. (B) Catechol oxidizes to quinone when an electric field is applied, triggering detachment. Reprinted with permission from Akram Bhuiyan et al.^120^ Copyright 2020 American Chemical Society.

Inspired by the catechol-containing adhesive proteins secreted by marine mussels, a catechol-derivative adhesive that can be deactivated through an electric field has been produced.^120^ The adhesive was synthesized through the photopolymerization of dopamine methacrylamide containing a catechol moiety, methoxyethyl acrylate, and methylene bis-acrylamide. A Johnson–Kendall–Roberts contact mechanics test was performed in a pH 7.5 aqueous buffer to measure the work of adhesion using a titanium sphere as a probe and an electrode and a platinum wire as a counter electrode. At 0 V (Figure 4A), the maximum tensile load was 70 mN. After 1 min at 9 V, the work and strength of adhesion reduced by 96% and close to 100%, respectively.

This reduction in adhesion^120^ is explained by the fact that the electric field induces water electrolysis, producing hydroxyl ions near the cathode and protons near the anode. Their presence changes the pH and oxidizes catechol to its quinone form characterized by poor adhesive properties (Figure 4B). It is worth noticing that elevated voltages result in irreversible oxidation due to cross-linking, although additional chemical protection might overcome this. An electroresponsive adhesive such as this may be useful in electronic devices by producing other catechol-based materials with tunable responsiveness and mechanical properties.

Electroadhesion has been applied to various chemical architectures. For instance, a new type of electrically responsive adhesion based on ionoelastomers of opposite polarity that can function at voltages of around 1 V has been reported.^121^ An ionoelastomer is a soft, dry conducting polymer network formed by an ionic liquid monomer cross-linked into an elastomer. The interface between two ionoelastomers is known as an ionic double layer (IDL), and it is the voltage drop across it that controls the adhesion between the complementary ionoelastomers. In this work, highly cross-linked networks of 1-ethyl-3-methyl imidazolium poly[(3-sulfopropyl) acrylate] (ES) and poly[1-(2-acryloyloxyethyl)-3-butylimidazolium]bis(trifluoromethane) sulfonimide (AT) were reinforced with 2.5 wt % fumed silica particles, and microporous-layer carbon electrodes were embedded in each layer.

To test electroadhesion, two cross-cylindrical samples were brought into contact for 30 s and then separated at a fixed voltage.^121^ ES/ES and AT/AT homojunctions were irresponsive to voltage. In contrast, the ES/AT heterojunction showed an important variation in the peak separation load (*P*_peak_) and critical strain energy release rate (*G*_C_) as a function of voltage. At 0 V, *P*_peak_ was 50 ± 3 mN and *G*_C_ was 1.1 ± 0.1 J m^–2^. Under a reverse bias of −2 V, *P*_peak_ was 170 ± 2 mN and *G*_C_ increased to 3.6 ± 0.1 J m^–2^, 4 times greater than the values recorded for ≥0.5 V.^121^

**Figure 5:**
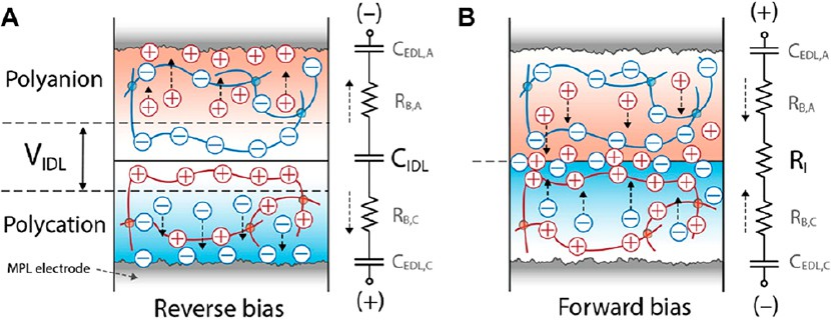
Schematic representations of a ionoelastomer junction operated under (A) reverse bias and (B) forward bias. Reprinted with permission from Kim et al.^121^ Copyright 2020 Wiley-VCH.

Further tests determined that these ionoelastomers can be switched on and off at ±1 V and can withstand shear stresses of up to 5 kPa.^121^ It was concluded that when the electroadhesive is exposed to a reverse bias (with the polyanion connected to the anode and the polycation connected to the cathode), mobile ions are pulled away from the IDL region, providing a build-up of excess fixed charges that leads to electrostatic adhesion (IDL behaves as a capacitor). When subjected to a forward bias, mobile ions are pushed toward the IDL, leading to the annihilation of the electric field across the IDL and hence to debonding (IDL behaves as a resistor).^121^ This is shown in Figure 5A,B.

**Figure 6:**
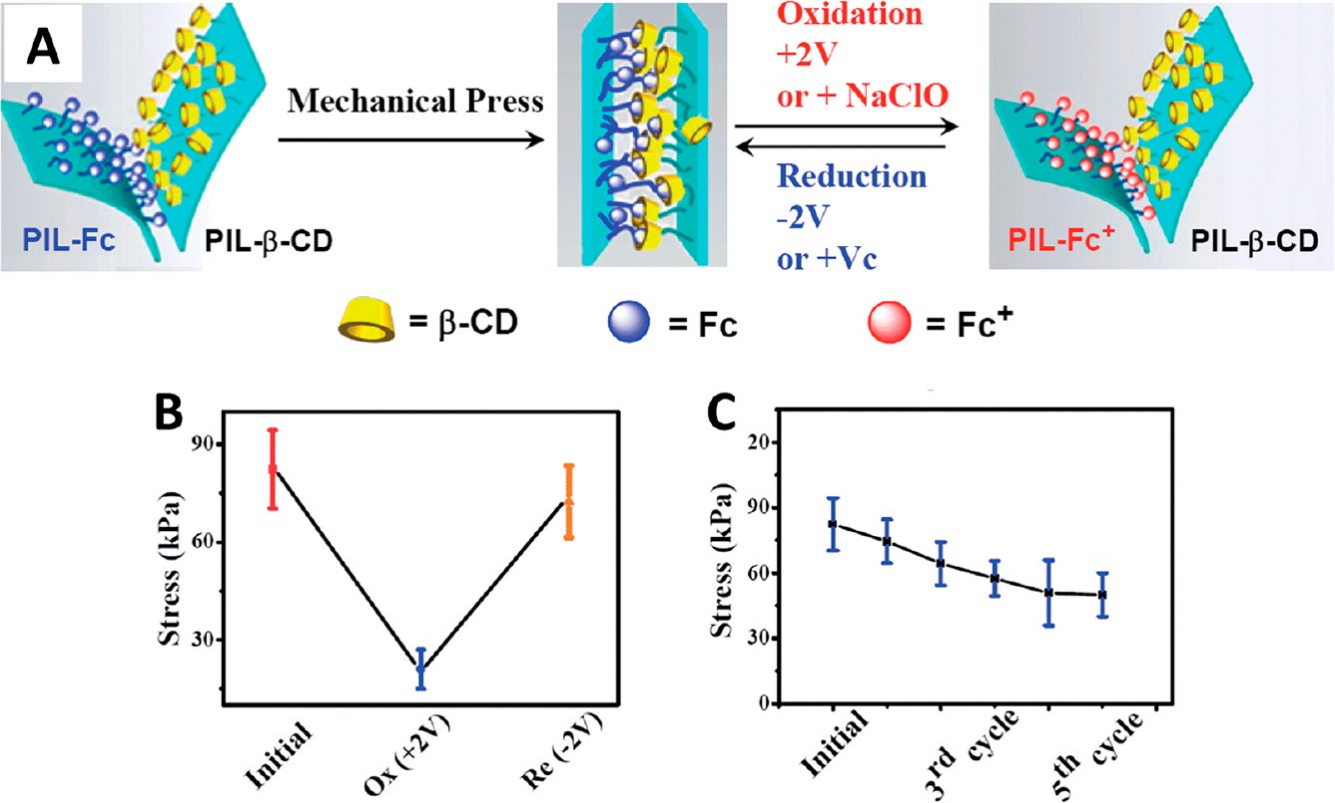
(A) Schematic representation of the bonding–debonding strategy for PIL-CD and PIL-FC membranes. (B) Reversibility by electrochemical means. (C) Reversibility by electrochemical means during five cycles. Reprinted with permission from Guo et al.^122^ Copyright 2014 Royal Society of Chemistry.

The interactions between host–guest supramolecular structures have also been studied to achieve electrically controlled adhesion. Among them, complexation between β-cyclodextrin (CD) and ferrocene (Fc) derivatives has been of interest because they can be reversibly associated through the redox state of Fc by either a redox agent or an electrochemical potential. Polymer velcros made of two functionalized poly(ionic liquid)s (PIL) were synthesized, one of which was conjugated with CD (PIL-CD) as a host and another modified with Fc (PIL-Fc) as a guest.^122^ These membranes adhere to each other under mechanical compression under dry or aqueous conditions through the molecular recognition between the host–guest pairs (Figure 6A).^122^

Adhesion experiments were performed in samples with a contact area of 0.4 × 0.8 cm^2^ by first adhering PIL-CD and PIL-Fc under a mechanical pressure of 400 g weight plates for 30 min at 30 °C.^122^ Samples were strong enough to withstand 100 g for over 3 h in dry and aqueous (neutral, acidic, and basic solutions and artificial seawater) environments. The lap shear adhesion strength of the PIL-CD/PIL-Fc couple under dry conditions was about 80 kPa. After five cycles of fastening and unfastening, ∼30% of the shear strength was lost. However, it could still withstand a 200 g weight for more than 2 h in all environments. The reversibility of the PIL-CD/PIL-Fc couple was tested under voltages of +2 V and −2 V. At +2 V, PIL-Fc oxidized, and under a negative voltage, it reduced. It was found that adhesion drastically decreased during the oxidized state, while it recovered up to 90% the original measurement after reduction and up to 59% after five cycles (Figure 6B,C).^122^

Furthermore, reversibility under redox conditions was also evaluated in NaClO where Fc was oxidized to Fc^+^.^122^ The adhesive behavior was similar to that of electric reversibility. PIL membranes have potential applications due to their tunability (e.g., host–guest moieties concentration and ratios) and mechanical properties (e.g., flexibility and reversibility).

A novel switchable adhesive with potential applications in climbing robots, sensors, and microfluidic devices has been demonstrated.^123^ Here, an N-doped graphene interface was produced through chemical vapor deposition, capable of tuning its adhesion according to an external electrical signal. When graphene is doped with nitrogen, it wrinkles and produces a nanotexture that significantly increases surface compliance. Adhesion measurements were performed using an atomic force microscope (AFM) by approach–retraction–approach cycles of the AFM tip covered with N-graphene and a silica microsphere at different humidity levels and electrical potentials. It was found that the adhesive force increased with both the voltage and humidity before reaching a maximum adhesive force at 15 V and 84% relative humidity, ∼6 times greater than at 0 V. The adhesive force could return to its normal values after 10 s after turning off the electric bias.

**Figure 7:**
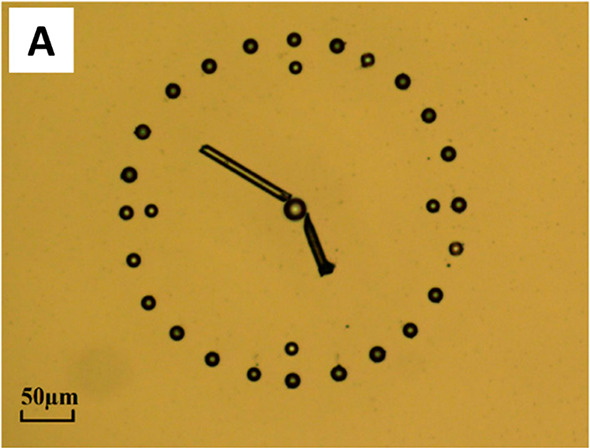
Manipulation of microparticles and microfibers. Reprinted with permission from Wan et al.^123^ Copyright 2019 American Chemical Society.

Such a change in adhesion is related to the fact that environmental moisture is collected within the nanotextured surface of N-graphene that can form hydrogen bonds with the target substrate. To prove the practical application of this material, an N-graphene pad was prepared to pick up, transport, and drop off a series of microparticles, debris, and microfiber segments (Figure 7). In particular, silica and polystyrene microsized objects could be precisely manipulated to produce patterns under DC and AC electric biases.^123^ In addition, it was found that repeated attachment and detachment cycles showed no decreased behavior even after 50 000 cycles.^123^ Though this was only a small-scale demonstration, it provided a fundamental understanding of an adhesive interface driven by noncovalent bonds, which could find application in a variety of fields from sticky tapes to microelectromechanical systems or even drug delivery.

On-command degradation could be advantageous in the development of dismantlable adhesives. In particular, the degradation of cross-linked polymers would be a means of achieving debonding. Electroactive cross-linkers may have a degradation mechanism that leads to the scission of bonds upon reduction at cross-linking sites triggered by an electric current. However, this area remains fundamentally theoretical as no experimental data has been published.^111,119^

Electroadhesion represents a simple strategy to produce reversible adhesive platforms in applications where conductivity is present. The goals here are to achieve debonding at a low voltage (10–50 V) and to decrease the detachment times.^100,117^ However, an ideal electroadhesive for a zero-waste industry would require the means to remove the material from the substrates and reprocess them or biodegrade them, for instance, through the development of biobased electroadhesives or electroactive cross-linkers from natural sources.

## Chemically Dismantlable Adhesives

The third route to producing dismantlable adhesives relies on the inclusion of chemically active moieties that react to an external stimulus. Such moieties are susceptible to degradation (cleavage),^124,125^ cross-linking,^125,126^ and isomerization^126,127^ or lead to a phase change.^127^ External cues refer to the need for a substance or condition that induces a change in the adhesive. Two of the most relevant adhesives of this kind are the thermally triggered and phototriggered mechanisms. Other lesser-studied systems include magnetically and electrostatically induced bonding and debonding.

### Thermally Triggered Dismantlable Adhesives

Reversible adhesion can be induced by thermodynamic changes. In particular, phase changes (e.g., from solid to liquid) are the most common mechanism choice for thermally triggered adhesives because these transitions are well understood.^127^

A recent example of a thermally triggered reversible adhesive for biomedical and industrial applications comprises an adhesive interfacial layer and a thermoresponsive adhesive matrix (TRP).^128^ Two types of systems were defined: gelatin gel was selected for warm release glues as it liquifies upon heating, and poly(*N*-isopropylacrylamide-*co*-butyl acrylate) (PNB) was chosen for cool release glues. Both the gelatin and the PNB displayed a strong interaction with tannic acid (TA), the adhesive interfacial layer.

Adhesion studies were performed *in vitro* on porcine skin.^128^ First, TA was deposited on the surface followed by the TRP. For PNB, a maximum work of adhesion of more than 200 J m^–2^ at 32 °C was observed with a drastic decrease when the temperature was lowered to 30 °C. For gelatin, a value of over 15 J m^–2^ was registered at 22 °C, with a reduced adhesion at 37 °C. Furthermore, the reversibility of this system was tested *in vivo* in ocular tissue and confirmed that PNB-based systems have the potential to treat penetrating injuries. Overall, bonding between TA and tissue occurs through electrostatic interactions while adhesion between TRP and TA is based on hydrophobic interactions and hydrogen bonding.

Another example of a thermoresponsive system involves a polycarbonate pressure-sensitive adhesive from propylene oxide (PO), glycidyl butyrate (GB), and CO_2_.^129^ The sample containing a 56:44 GB/PO molar ratio displayed the highest peel force of 4.9 ± 0.4 N cm^–1^ with glass and paper as substrates. This value is superior to that of Scotch tape (2.1 ± 0.2 N cm^–1^) and similar to that of duct tape (4.1 ± 0.5 N cm^–1^).

**Figure 8:**
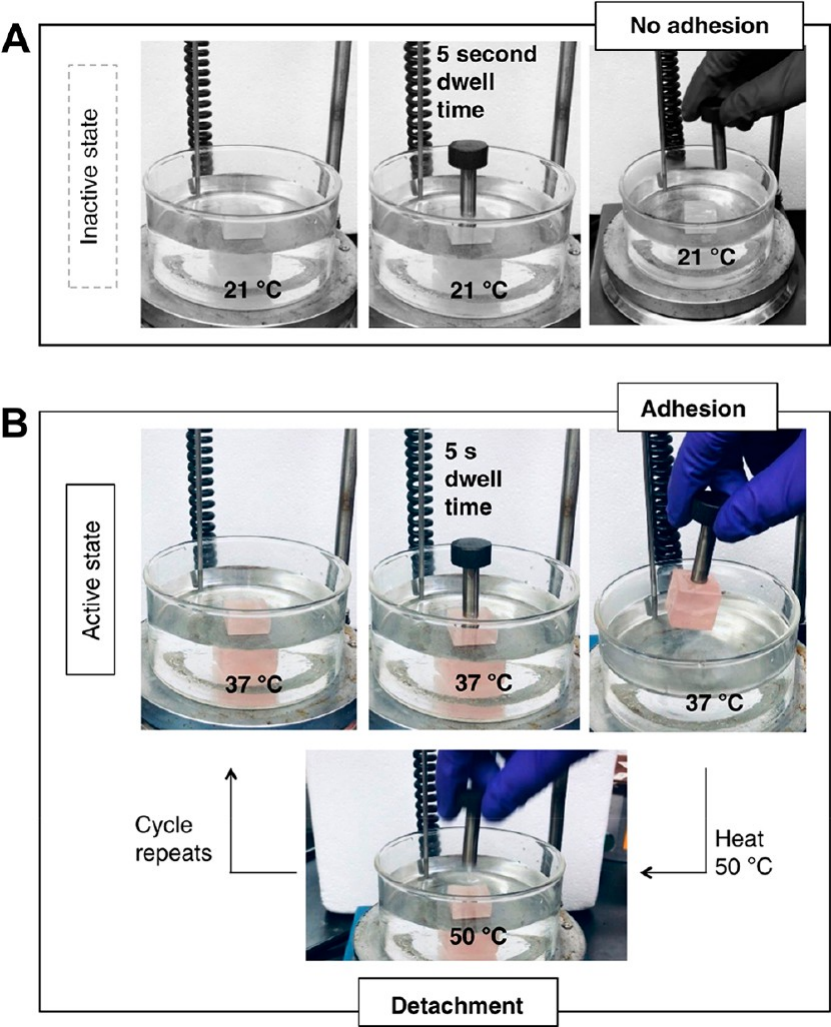
Adhesive system with thermoresponsive bonding and debonding. (A) Inactive adhesion at 21 °C. (B) Active adhesion at 37 °C and deactivation at 50 °C. Reprinted from Beharaj et al.^129^ Creative Commons Attribution 4.0 International License.

The temperature responsiveness was evaluated by measuring the adhesion energy at 37 and 50 °C under dry and wet conditions.^129^ The optimized sample displayed a dry tack strength (with 1 N of applied axial pressure and 5 s of dwell time) of 9.0 ± 1.8 N at 20 °C, 30.4 ± 5.2 N at 37 °C, and 9.6 ± 1.6 N at 50 °C which is the desired bonding–debonding tack profile. This same trend was observed under wet conditions. This behavior was corroborated with an adhesive system formed by a glass cube (20 g) coated with the optimized sample to which a steel rod was attached underwater. At 21 °C, adhesion did not occur, but when the temperature increased to 37 °C, the rod could pick up an object (Figure 8). Adhesion was deactivated as the temperature increased to 50 °C.

It was concluded that the change in viscosity produced by the temperature trigger was fundamental to regulating van der Waals interactions between polymer chains and in the adhesive–substrate interface, consequently reversing adhesion. Cytotoxicity was seen to be limited in the tests performed, making these adhesives useful for consumer goods and medical devices.^129^

An interesting approach to thermal reversibility is the thermally induced decomposition that degrades the polymer backbone and, in some cases, produces gases that weaken the adhesive bond at the interface.^111^ These can be, for instance, the linkage between isocyanate and hydroxyl or amine functional groups from urethanes and substituted urea bonds, the inclusion of peroxide bonds in the polymer backbone or as cross-linking points, and rubber compositions showing thermoreversible reactions, among others.^111,130^

A particular reversible adhesive system based on a Diels–Alder stimuli-responsive moiety that can degrade upon heating was reported.^124^ Here a polymer adhesive was synthesized with a dimethacrylate cross-linker including two thermally sensitive hetero-Diels–Alder moieties (DiHDA-linker) through free radical polymerization. When heated to above 80 °C, the presence of visible red dithioester species in the degradation products could be quantified through UV–vis spectroscopy. Thermal degradation was evaluated, concluding that at 100 °C debonding equilibrium was achieved in under 3 min. Furthermore, the pull-off force was measured at 23 and 80 °C in dental crowns. The DiHDA linker or a conventional dimethacrylate cross-linker was copolymerized with *n*-butyl methacrylate. Both systems showed good adhesion at 23 °C; however, the one containing the DiHDA linker lost 94% of its adhesion at 80 °C. The conventional system lost only 42%.

Thermoadhesion is one of the most promising strategies due to the variety of materials that can display phase changes. However, response times are often slow, requiring further research on new formulations. In addition, several of the chemical structures require molecular design and more complex synthesis processes, which would limit their industrial application. The main potential of these materials relies on the fact that the thermal trigger can come from other stimuli or can be used in conjunction with other strategies.

### Phototriggered Dismantlable Adhesives

Light-curable adhesives have been widely used for manual and assembly line processes, which is why using light to reverse adhesion has been studied. Typically, the peel adhesion and probe tack are directly related to the number of photoreactive groups and the applied UV radiation (time and intensity).^130−132^ Debonding on demand can be achieved by exposure to radiation in the UV–vis range through two mechanisms: photoinduced degradation or phototriggered phase changes. The principal advantage of photoirradiation is that it allows precise spatial control over the removal of adhesives.^130^

Photodegradation consists of the scission of the polymer molecules that lowers their molar mass and therefore affects their mobility, entanglement, and the overall mechanical properties of the network.^130,133^ Adhesives displaying such behavior require the introduction of photocleavable functionalities such as *o*-nitrobenzyl compounds, *p*-hydroxyphenacyl, and coumarin-4-yl-methyl.^134^

A dismantlable adhesive based on a thermo- and photocleavable layer containing an anthracene photodimer has been demonstrated.^135^ Anthracene has been widely characterized before and is known to form reversible covalent bonds through photodimerization and photocleavage processes. This switchable anthracene layer was formed in the adhesive interface and increased the adhesion strength due to the formation of covalent bonds between the substrate and the adhesive. When exposed to 254 nm radiation, the adhesion strength was reduced by 33%, as measured in a peel test. Debonding also occurred upon heating to 180 °C for 1 min even more effectively as the adhesion strength decreased by 60%. The detachment process required 4–10% of the energy of other phototriggered dismantlable adhesives. This approach can be useful for a variety of substrates and adhesives, and substrates remain reusable as failure occurs in the interface. However, the main challenge is related to the preparation of the molecular layer on the surface as this procedure has been performed only as a proof of concept.

A phototriggered adhesive based on cross-linked poly(olefin sulfone) has also been prepared.^136^ The adhesive was synthesized from cross-linkable poly(olefin sulfone), a polycarbodiimide cross-linking agent, and a photobase generator. Quartz plates were bonded with the adhesive, and their adhesive strength was measured and compared with that of commercially available epoxy resin Araldite rapid. The photoadhesive showed superior strength due to the high polarity of the main chain containing carboxylic acid groups and the cross-linking points containing *N*-acylurea groups.

**Figure 9:**
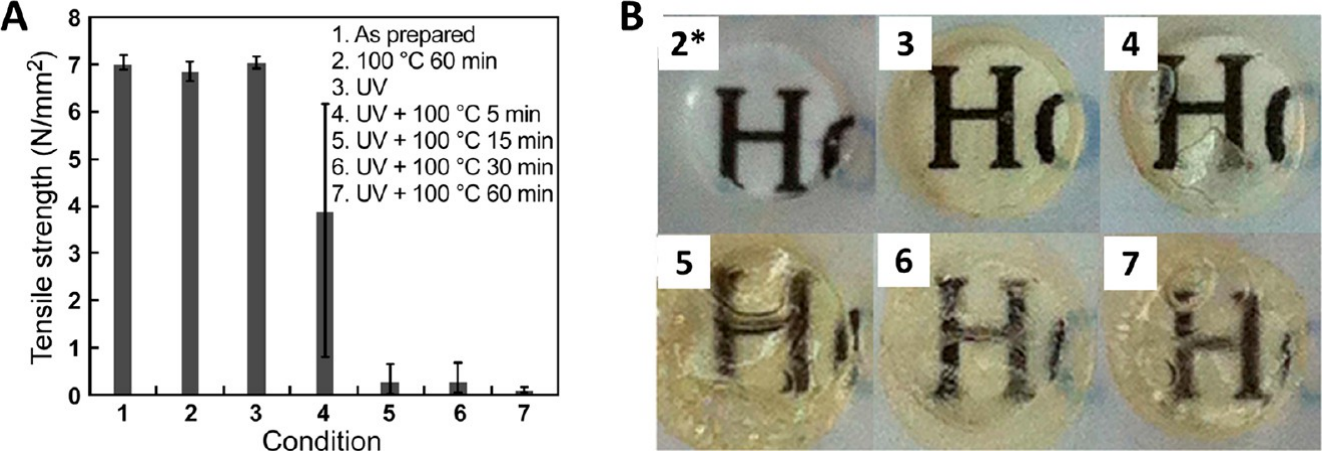
(A) Strength of phototriggered adhesives under different conditions. (B) Photographs of the adhesive after being exposed to conditions 2*–7 and 2*: after holding at 100 °C for 30 min. Reprinted with permission from Sasaki et al.^136^ Copyright 2016 American Chemical Society.

Detachment was studied under UV light and a UV plus temperature trigger,^136^ and the results are shown in Figure 9A,B. It was found that samples exposed to 254 nm UV irradiation and then heated to 100 °C for 60 min showed an adhesive strength of close to zero. After UV exposure, the adhesive became yellow because of the production of nitrosobenzaldehyde. Also, when heated, gaseous products were generated from the decomposition of poly(olefin sulfone), further promoting separation.

Azo compounds are widely employed for phototriggered phase changes, which occur when a polymer network becomes liquid or solid under radiation. Azobenzene behavior is based on its *trans*–*cis* photoisomerization and exhibits what is known as photoliquification or photomelting.^126,137^ During this process, the change in configuration influences chain mobility and packaging, inducing a solid-to-liquid transition.

Recently, the synthesis of an ABA triblock copolymer containing a poly(azobenzene methacrylate) moiety (A block) and poly(2-ethylhexyl methacrylate) (B block) has been described.^138^ Because B is a soft middle block without the azo component, this polymer was flexible and enabled film production. As mentioned, azobenzene changes its viscosity under radiation. It softens in 365 nm UV light and hardens in 520 nm green light. To study this behavior, two glass substrates were bonded with the adhesive and their shear strengths were measured under UV and green light radiation. The maximum shear strength was 1.5–2.0 MPa with a decrease to 0.5–0.1 MPa under UV irradiation. Bonding and debonding could be repeated without any loss in strength. Mechanical properties under UV irradiation were similar to those of conventional PSA and hot-melt adhesives.^49^ Phototriggered adhesives of this type could be useful for biomedical applications or substrates that cannot withstand heat.

The reversibility of adhesion is based on the chemical composition, UV–vis wavelength, and time of exposure. However, these applications are limited because of the penetration depth of light, thus restricting the materials (e.g., opaque materials) that can be bonded through this strategy. In addition, most of the chemical synthesis requires specialized processes that cannot be readily scaled-up with the current processing lines and compound availability.

### Other Dismantlable Adhesives

Apart from the dismantlable adhesives described above, there are other mechanisms that have just recently been described. The most relevant ones are those triggered by a magnetic field and those based on electrostatic interactions. These have been developed to overcome some of the obstacles other mechanisms display.^126^

Magnetic fields have the advantages of being noncontact, easily tailored, controlled, and generated through permanent or electromagnets.^139^ These characteristics make them suitable to be used in reversible adhesive systems. The main requirement for these is that they must include elements displaying magnetic properties (e.g., superparamagnetism) usually based on iron, aluminum, cobalt, or nickel.^126,130^

Magnetic additives can be used to induce degradation under an oscillating magnetic field. However, this degradation is a consequence of hysteresis heating, a phenomenon observed for superparamagnetic nanoparticles.^126,140^ The heat may act following the mechanisms described for thermotriggered dismantlable adhesives by melting a thermoplastic matrix or activating expansion agents.^100,130^ The inclusion of paramagnetic species is affordable and poses additional advantages, such as the improvement of the mechanical properties. However, the application of a magnetic field would limit the dimensions of the pieces to be detached.

Some magnetically triggered dismantlable adhesives are biomimetic structures containing magnetic elements. Adhesion is controlled remotely, and a fast response is induced by modifying the surface topography.^126,130,141^ Research needs to be further developed on formulations and procedures suitable for scaling up the microfabrication processes.

Electrostatic adhesion is typically available when two oppositely charged polyelectrolytes attach to each other in the presence of water, leading to the formation of polyelectrolyte complexes.^142^ This kind of adhesion can be reversed by immersing the system in a salt solution or a solution of a particular pH as it is the protonation and deprotonation processes controlling the interaction between polyelectrolytes.^98,143−145^ Related to this phenomenon, if only one component is a polyelectrolyte, then pH-reversible adhesion may still occur through the control of hydrogen bonding sites.^146^ These systems are of particular interest because debonding occurs in an aqueous environment and is useful for underwater applications.

Although the use of magnetic fields or electrostatic interactions would be convenient to be applied to a variety of joint materials, so far only the science behind the reversibility of this kind of adhesion has been studied. For these approaches to be successful, the production of such surfaces must be scalable, which is the main limitation for many of the strategies described before.

The development of dismantlable adhesives will be fundamental for the success of zero-waste industries. Table 3 summarizes the reversibility mechanisms that have been developed up until the present. Different approaches can be followed to fulfill the requirements of particular applications, with the aid of both basic and advanced chemistries. However, approaches for adhesive removal consistent with zero-waste principles are yet to be found, as solvent-based and mechanical methods are still used.

**Table 3:** Dismantlable Adhesives and Their Mechanisms and Chemistries

adhesive	mechanism	chemistry
hot-melt adhesives	■ softened when heated	■ conventional thermoplastics
adhesives containing expansion agents	■ blowing agents that expand when heated	■ conventional thermally expandable microcapsules: thermoplastic polymer shell and a core of a hydrocarbon with a low boiling point
		■ thermally expandable fillers (e.g., doped graphite, pearlite, mica, etc.)
electroadhesives	■ reversible when an electric potential is generated in the interface of adhesion	■ conductive substrates
		■ adhesive polymer displaying ion mobility or redox reactions
thermally triggered adhesives	■ reversible when a change in temperature produces	■ thermoresponsive polymers (e.g., PNIPAM)
	- thermally induced phase changes	■ polymers with thermally degradable moieties (e.g., peroxide bonds)
	- thermal degradation	
phototriggered adhesives	■ reversible by exposure to radiation due to	■ photoisomers (e.g., azo compounds)
	- photoinduced phase changes	■ polymers with photocleavable functional groups (e.g., anthracene, *o*-nitrobenzyl, etc.)
	- photodegradation	
other adhesive systems	■ magnetically triggered reversibility due to hysteresis heating	■ hot melts, adhesives containing expansion agents or phototriggered adhesives embedded with superparamagnetic nanoparticles (e.g., Fe, Al, etc.)
	■ electrostatic adhesion reversed by protonation and deprotonation processes	■ polyelectrolytes

## Conclusions

The use of adhesives based on natural materials was the norm before the 20th century, but the resurgence of these due to their sustainability currently faces the limitations imposed by the demands that are easily met by their synthetic counterparts. Though biomaterials can be modified to improve their adhesive and resistance properties, these modifications often require the inclusion of nonrenewable compounds, reducing the environmental friendliness of the process and final adhesive.

From a sustainability perspective, the most attractive approach would be the design of macromolecular structures from natural raw materials. The combination of natural monomers and biopolymers to create materials capable of displaying adhesive reversibility would be even more appealing. Furthermore, natural or enzyme-induced degradation would ensure that these adhesives fulfill the requirements of a zero-waste industry.

Considering the current state of adhesion technology, which is widely based on synthetic materials, an ideal adhesive for a zero-waste industry would be one in which adhesive is recovered and recycled. However, recycling remains the most elusive process: first, because removing the adhesive from the substrate can be problematic and second, because of its composition. A possibility would be to use existing waste materials to produce adhesives. So far, little research has been performed in this area.

The design for a circular economy should be driven away from new monomers with particular moieties because these are usually obtained in small quantities and often require highly specialized synthesis procedures. A more realistic approach would be to use existing initial materials and modify them, as required, through existing techniques that can be scaled up if necessary. Polymers, copolymers, blends, and composites (including those with organic and inorganic phases) could be developed to be applied in more than one field, rather than for niche applications.

For a new adhesive technology to be successful, if it is developed from natural materials or as a dismantlable adhesive, not only should its properties be appropriate but also the cost of the adhesive system, including reagents, processing equipment, and dismantling tools, must be comparable to those of current adhesive technologies. This would exclude initial compounds that require previous and costly modification or extensive purification procedures. In addition, the use of current industrial production processes would be advantageous for a smooth integration into the existing systems.

Design within the 3R paradigm is fundamental for integrating adhesives into a zero-waste system. The new generation of adhesives should be developed from renewable materials, waste products, or the substitution of harmful reagents with less dangerous ones. Debondable adhesives are the most promising alternative as they allow the reintegration of the joint parts into the production flow, hence decreasing the amount of solid waste that would end up in oceans and landfills. A step toward a more sustainable adhesive industry would be the use of biobased materials to produce reversible systems and the development of either adhesive recovery technologies or degradable products.

## Abbreviations

VOCs: volatile organic compounds
IA: itaconic acid
MCC: microcrystalline cellulose
DAC: dialdehyde cellulose
CNC: nanocrystalline cellulose
PVA: poly(vinyl acetate)
PEGMA: poly(ethylene glycol) methacrylate
PGSu: poly(glycerol succinate)
PGM: poly(glycerol maleate) (PGM)
PSAs: pressure-sensitive adhesives
OLA: lactic acid oligomers
ESO: epoxidized soybean oil
PU: polyurethane
TEG: triethylene glycol
DBTDL: dibutyltin dilaurate
PLLA: poly(l-lactide)
PDL: poly(ε-decalactone)
RE: rosin ester
MMA: methyl methacrylate
TEM: thermally expandable microcapsule
AN: acrylonitrile
MA: methacrylate
EG: expandable graphite
IDL: ionic double layer
ES: 1-ethyl-3-methyl imidazolium poly[(3-sulfopropyl) acrylate]
AT: poly[1-(2-acryloyloxyethyl)-3-buthylimidazolium]bis(trifluoromethane) sulfonimide
CD: β-cyclodextrin
Fc: ferrocene
PIL: poly(ionic liquid)
AFM: atomic force microscope
TRP: thermoresponsive adhesive matrix
PNB: poly(*N*-isopropylacrylamide-*co*-butyl acrylate)
TA: tannic acid
PO: propylene oxide
GB: glycidyl butyrate
